# Mirror Movements and Myelomeningocele: Report of A Single Case and Review of Literature

**Published:** 2013

**Authors:** Ibtihel RéBAI, Hanene BENRHOUMA, Ichraf KRAOUA, Cyrine DRISSI, Mohammed Ben HAMMOUDA, Neziha GOUIDER-KHOUJA

**Affiliations:** 1 Department of Child and Adolescent, Neurology and Movement Disorders & Botulinum Toxin Consultation, Mongi Ben Hmida Institute of Neurology, Tunis, Tunisia; 2 Department of Neuroradiology, Mongi Ben Hmida Institute of Neurology, Tunis, Tunisia

**Keywords:** Mirror movements, Cervical myelomeningocel, Diffusion tensor imaging

## Abstract

**Objective:**

Mirror movements (MM) have been described in several pathological conditions. Their association with neural tube defects is rare, and only 5 cases have been reported in literature to date. We report on a case of MM associated with cervical myelomeningocele, and we discuss the diffusion tensor imaging findings and the underlying mechanism.

## Case report

**Fig 1:**
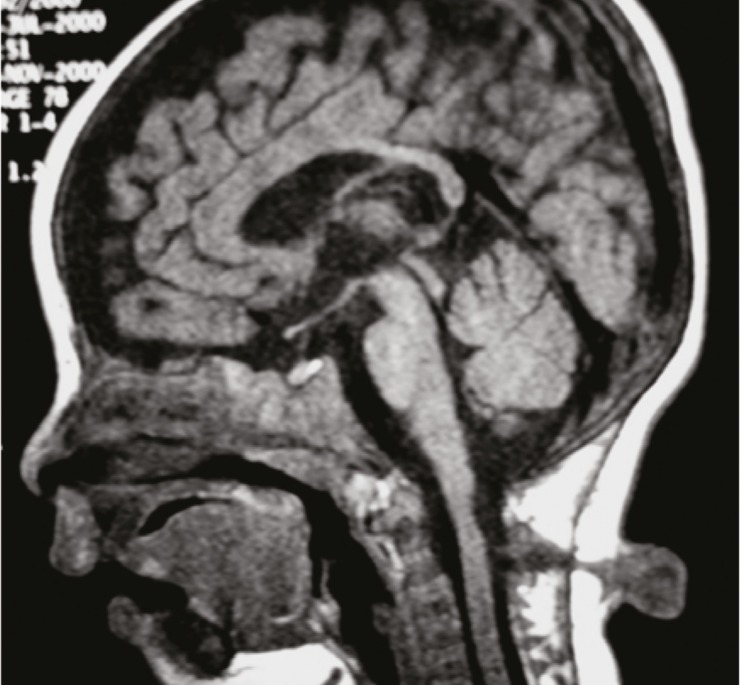
Cervical sagittal T1 weighted MRI shows cervical myelomeningocele at the C2-C3 level

An 11-year-old right-handed boy was born to non consanguineous parents, with no family history. At birth, he was diagnosed with cervical myelomeningocele (Fig1) and operated at the age of 7 months with no immediate postoperative complication. Five months later, his parents noticed that voluntary movements of one hand was unintentionally reproduced by the other hand. Developmental milestones were normal.

At the age of 6 years, he complained of difficulties in bimanual activities. He was referred to our “Movement Disorders and Botulinum Toxin” consultation for evaluation of movement disorders in hands. Neurological examination showed bilateral involuntary synkinetic imitative movements in one hand, occurring with all voluntary movements of the opposite hand, concluding to hand’s MM. Amplitude of the intended movement was higher than that of the mirroring hand. He had also a right trapezium muscle atrophy and pyramidal syndrome in all four limbs. Sensory examination was normal.

Cerebral and spinal MRI at the age of 7 years was normal. EMG and NCV revealed mild neurogene changes in trapezium, supraspinatus and infraspinatus nerves, bilaterally.

**Fig 2:**
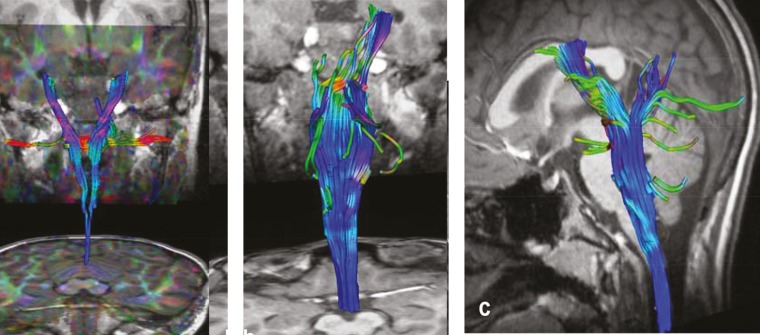
Diffusion tensor imaging of brainstem and cervical spine

Diffusion tensor imaging (DTI) of the brainstem and the cervical spine was performed. No obvious cortico-spinal tract decussation in lower medulla oblongata was observed. Cervical DTI was normal (Fig. 2 a,b,c,).

Coronal (a,b) and sagittal (c) sections of the brainstem. No evidence of cortico-spinal tract decussation in lower medulla oblongata is noticed. Cervical DTI is normal (c)

## Discussion

This case report illustrates a rare association of MM and cervical myelomeningocele. The association of MM with congenital neural tube defects is rare. Only five cases have been reported in literature (Table 1) (3-7). Our patient showed clinical and paraclinical similarities with the reported cases. In fact, MM were described to have a childhood onset (4,6,7). They were usually seen in upper limbs (distal parts of hand) and reproducing all movements of the opposite side (3,4,6,7). Other clinical features (pyramidal syndrom, sensory impairment) were reported and were related to congenital neural tube defects (3,4,6). All patients had neuroimaging investigations (CTscan, cerebral and spinal MRI) in order to determine the type of the neural tube defect and the eventual spinal cord involvement (3-7). However, DTI was not performed in these cases. Some of them underwent an electromyographic study or a transcranial magnetic stimulation (TMS) to better understand the underlying mechanisms (3-5,7). Pathophysiological mechanisms of MM vary according to the pathological condition. Overactivation of ipsilateral corticospinal tract and abnormal transcallosal inhibitory connexions are the major hypotheses. In cases of neural tube defects, the underlying mechanism corresponds to damage in the corticospinal tract decussation in lower medulla oblongata, which results in alternative less specific bilateral spinal pathways (2). Activation of the uncrossed fibers will generate ipsilateral movements reproducing the intentional movements in the opposite side. Closeness of lower medulla oblongata to the cervical myelomeningocele in the reported cases is highly suggestive of a more complicated defect with uncrossed corticospinal tract. This hypothesis is supported by pathological findings in cases of Klippel Feil syndrome presenting with cervical myelomeningocele, mirror movements, and uncrossed corticospinal fibers (8,9). Nevertheless, Klippel Feil syndrome was not reported in these five cases and excluded in our patient. Actually, using advanced radiological functional techniques, such as DTI may be helpful in detecting corticospinal tract pathways in vivo, without resorting to pathological examinations. In this case report, no evidence of corticospinal tract decussation was noted on DTI of lower medulla. This could be a corroborating evidence of the presumed pathophysiological mechanism. However, DTI with high angular resolution may be more efficient to demonstrate uncrossed pathways (10,11). Unfortunately, no effective treatment is currently available (12).

**Table 1:** Reported Cases of Mirror Movements Associated with Cervical Neural Tube Defects

Authors, year	Number of cases	Gender	Age (years)	Age of MM onset	Clinical features	Neural tube defect nature
Forget et al., 1986	1	M	_	Adulthood (25 years)	MM, deep sensation impairment	Cervicodorsal meningocele
Odabasi et al., 1998	1	M	_	Childhood	MM	Cervical meningocele, anomaly at the posterior to the cervical spinal cord-medulla junction
Erdincler et al., 2002	1	F	18	Childhood	MM, broad-based unsteady gait, pyramidal syndrome	Cervical meningocele, spinal cord tethering
Erol et al., 2004	1	F	14	Childhood	MM, brisk tendon reflexes, recurrent meningitis	Cervical meningocele and dermoid sinus tract
Andrabi et al., 2008	1	M	3	Childhood	MM	Cervical myelomeningocele
Our case	1	M	11	Childhood (1 year)	MM, pyramidal syndrome, right trapezium hypotrophy	Cervical myelomeningocele

MM: mirror movements; M: male, F: female

**In conclusion,** our observation is exceptional based on the rare association of MM to cervical myelomeningocele. MM should be searched in every child with cervical myelomeningocele. DTI with high- resolution line may be a helpful tool to better understand the underlying mechanisms.
